# Left atrial appendage exclusion in atrial fibrillation

**DOI:** 10.3389/fcvm.2022.949732

**Published:** 2022-09-13

**Authors:** Guy Rozen, Gilad Margolis, Ibrahim Marai, Ariel Roguin, Eldad Rahamim, David Planer, Edwin Kevin Heist, Offer Amir, Ilgar Tahiroglu, Jeremy Ruskin, Moussa Mansour, Gabby Elbaz-Greener

**Affiliations:** ^1^Cardiovascular Center, Tufts Medical Center, Boston, MA, United States; ^2^Cardiac Arrhythmia Center, Massachusetts General Hospital, Harvard Medical School, Boston, MA, United States; ^3^Cardiology Division, Hillel Yaffe Medical Center, Hadera, Israel; ^4^The Ruth and Bruce Rappaport Faculty of Medicine, Technion, Haifa, Israel; ^5^Division of Cardiovascular Medicine, Baruch Padeh Medical Center, The Azrieli Faculty of Medicine in the Galilee, Bar-Ilan University, Safed, Israel; ^6^Department of Cardiology, Hadassah Medical Center, Jerusalem, Israel; ^7^Faculty of Medicine, Hebrew University of Jerusalem, Jerusalem, Israel; ^8^Department of Cardiology, Baku Health Center University, Baku, Azerbaijan

**Keywords:** left atrial, LAAC, structural intervention, appendage, devices, atrial fibrillation, stroke, prevention

## Abstract

Although oral anticoagulants (OACs) are first-line therapy for stroke prevention in patients with atrial fibrillation (AF), some patients cannot be treated with OACs due to absolute or relative contraindications. Left atrial appendage (LAA) exclusion techniques have been developed over the years as a therapeutic alternative for stroke prevention. In this paper, we review the evolution of surgical techniques, employed as an adjunct to cardiac surgery or as a stand-alone procedure, as well as the recently introduced and widely utilized percutaneous LAA occlusion techniques. Until recently, data on surgical LAAO were limited and based on non-randomized studies. We focus on recently published randomized data which strongly support an add-on surgical LAAO in eligible patients during cardiac surgery and could potentially change current practice guidelines. In recent years, the trans-catheter techniques for LAA occlusion have emerged as another, less invasive alternative for patients who cannot tolerate oral anticoagulation. We review the growing body of evidence from prospective studies and registries, focusing on the two systems which are in widespread clinical use nowadays: the Watchman and Amulet type devices. These data show favorable results for both Watchman and Amulet devices, setting them as an important tool in our arsenal for stroke reduction in AF patients, especially in those who have contraindications for OACs. A better understanding of the different therapeutic alternatives, their specific benefits, and downfalls in different patient populations can guide us in tailoring the optimal therapeutic approach for stroke reduction in our AF patients.

## Background

Atrial fibrillation (AF) is the most commonly encountered arrhythmia in clinical practice, associated with increased morbidity (1–3), mortality (4), and healthcare expenditures (5). AF increases the risk of ischemic cerebrovascular events by a factor of four to five (6) and accounts for up to 15% of strokes in persons of all ages and up to 30% of strokes in persons over the age of 80 years (7).

In patients with non-valvular AF, risk stratification using the well-validated CHA_2_DS_2_-VASc scoring system is guiding the therapeutic approach for stroke prevention (8–10). Oral anticoagulation (OAC), using vitamin K antagonists (VKA) and, in the last decade, direct oral anticoagulants (DOACs) in the non-valvular AF patients (i.e., no prosthetic mechanical valve or moderate-severe mitral stenosis), was proven to be very effective and is recommended for stroke prevention in AF patients with CHA_2_DS_2_-VASc risk scores of 1 (non-sex) and above (11–14).

However, some patients cannot be treated with OAC because of objective absolute or relative contraindications. The rationale for left atrial appendage (LAA) exclusion, usually as an alternative for anticoagulation, rests upon the thrombotic potential of the hemodynamically idle LAA. As shown in the past, most strokes in patients with AF result from thrombus formation in the LAA (15–17). In a review of 23 studies, when documented, thrombi were present in the LAA in 201 of 222 (91%) non-rheumatic AF patients and 254 of 446 (57%) patients with rheumatic AF (18).

For these reasons, surgical and transcatheter approaches have been investigated for the risk reduction of stroke by means of excluding or occluding the LAA (15). Several methods have been developed and used over the years to exclude the appendage: surgical excision (19–21), endocardial or epicardial suture during concomitant cardiac surgery (15–17, 22, 23), epicardial exclusion by stapling or clips (23–25), or endovascular occlusion by percutaneous devices (24, 26, 27).

Presently, real or perceived contraindications for OAC, typically related to concerns for bleeding hazards, are the primary motivation to expand the use of LAA occlusion techniques in high-risk AF patients. Although DOACs were shown to have a significantly improved safety profile compared with VKA, especially in regard to the feared complication of intracranial bleeding (28–32), severe bleeding under OAC therapy including DOACs is still a persistent and pertinent hazard, especially for patients with high bleeding risk. Our goal in this manuscript is to review the data regarding the use of LAA exclusion techniques including the contemporary data from the current era of anticoagulation therapy.

### The surgical techniques

Several surgical techniques were investigated over the years to excise or exclude the LAA from the circulation, hoping to reduce the risk of stroke in AF patients. In general, these can be divided into interventions during a concomitant cardiac surgical/MAZE procedure or a stand-alone, closed-chest procedures. Following the observation of LAA as a significant site of thrombi formation in patients undergoing valvular surgeries for rheumatic heart disease, Maden performed the first documented LAA excision in two patients with AF and rheumatic mitral valve in 1949 (33). Unfortunately, similarly to Maden's experience, Leonard, and Cogan, who followed, encountered a high rate of complications, especially neurologic complications (34). Later, echocardiography development and surgical techniques improvement reignited the interest in the surgical approach for stroke risk reduction in AF patients. Johnson et al. reported prophylactic LAA excision in 437 patients who underwent an open-heart surgery between 1995 and 1997, with a low rate of perioperative cerebrovascular accidents (19).

A different approach using LAA ligation instead of excision was developed to try and achieve its exclusion with a more accessible and less traumatic technique. In the beginning, these procedures were performed using epicardial or endocardial sutures. Studies showed suture ligation of the LAA to be frequently incomplete, leaving a communication that increased the risk of embolism (16, 35–37). Techniques using surgical staplers with (38) or without (39) excision of the LAA were introduced. However, stapler lines frequently bleed, and recanalization of the lumen was observed (38).

The early surgical literature on LAA closure consisted primarily of retrospective case series of patients who underwent LAA occlusion and later presented with new findings warranting transesophageal echocardiography (TEE) evaluation, resulting in a selection bias. In a randomized trial from 2005 that examined the efficacy of prophylactic occlusion of the LAA in the prevention of stroke, Healey and his colleagues studied 77 patients with known AF or risk factors for AF, undergoing CABG (w/o concomitant valvular surgery), randomized to undergo LAA occlusion (2:1 ratio, favoring occlusion), using sutures or stapling device (17). The success rate was 45% for sutures and 72% for stapling technique. Two periprocedural strokes occurred in the study group (vs. none in the control group), and no late cerebrovascular events were recorded in both groups during 13 ± 7 months follow-up period (17, 20, 40).

To offer a less invasive LAA occlusion, Blackshear et al. described successful thoracoscopic obliteration of the LAA using a stapled or snare technique (41). The procedure was performed on 15 patients who suffered from AF, high risk for stroke (majority with a history of CVA), and a documented contraindication for anticoagulation. During a mean follow-up of 42 months, two strokes and two incidences of deaths were recorded. Of the 11 patients with previous thromboembolic events, a stroke rate of 5.2%/year was lower but without statistical significance compared to the aspirin-treated historical cohort of patients from the SPAF trials (13%/year) (42). Another group from Japan showed high safety and efficacy of thoracoscopic stand-alone LA appendectomy in 30 patients with non-valvular AF, previous stroke, and contraindication to anticoagulation (43). Despite the discontinuation of oral anticoagulation, no patient experienced cerebrovascular events during a mean of 16 months follow-up.

Recently, novel LAA exclusion devices were introduced to exclude the LAA from circulation. The Atriclip Device System (Atricure, Inc., West Chester, Ohio), used during open cardiac surgery or a thorascopic procedure, was shown to have promising results, yet larger trials with longer follow-up are needed to assess its safety and long-term efficacy (44–47). Another observational study assessed the efficacy of surgical LAA exclusion vs. standard procedure among older patients (age>65) with AF undergoing cardiac surgery (48). Using the Society of Thoracic Surgeons Adult Cardiac Surgery Database registry, the authors identified 10,524 patients who underwent cardiac surgical procedures, 37% of whom had concomitant surgical LAA exclusion. Their analysis showed a 37% decrease in thromboembolic events among patients who had LAA exclusion in the 3-year follow-up (48).

In a large meta-analysis of 22 studies (*n* = 280,585) which included patients who underwent cardiac surgery of any kind, the performance of LAA exclusion was associated with lower rates of stroke and embolic events peri-operatively and in >2 years follow-up period. Interestingly, while peri-operative mortality rates were similar, a long-term survival benefit was observed for patients who had concomitant LAA exclusion during cardiac surgery (49).

**Figure 1:**
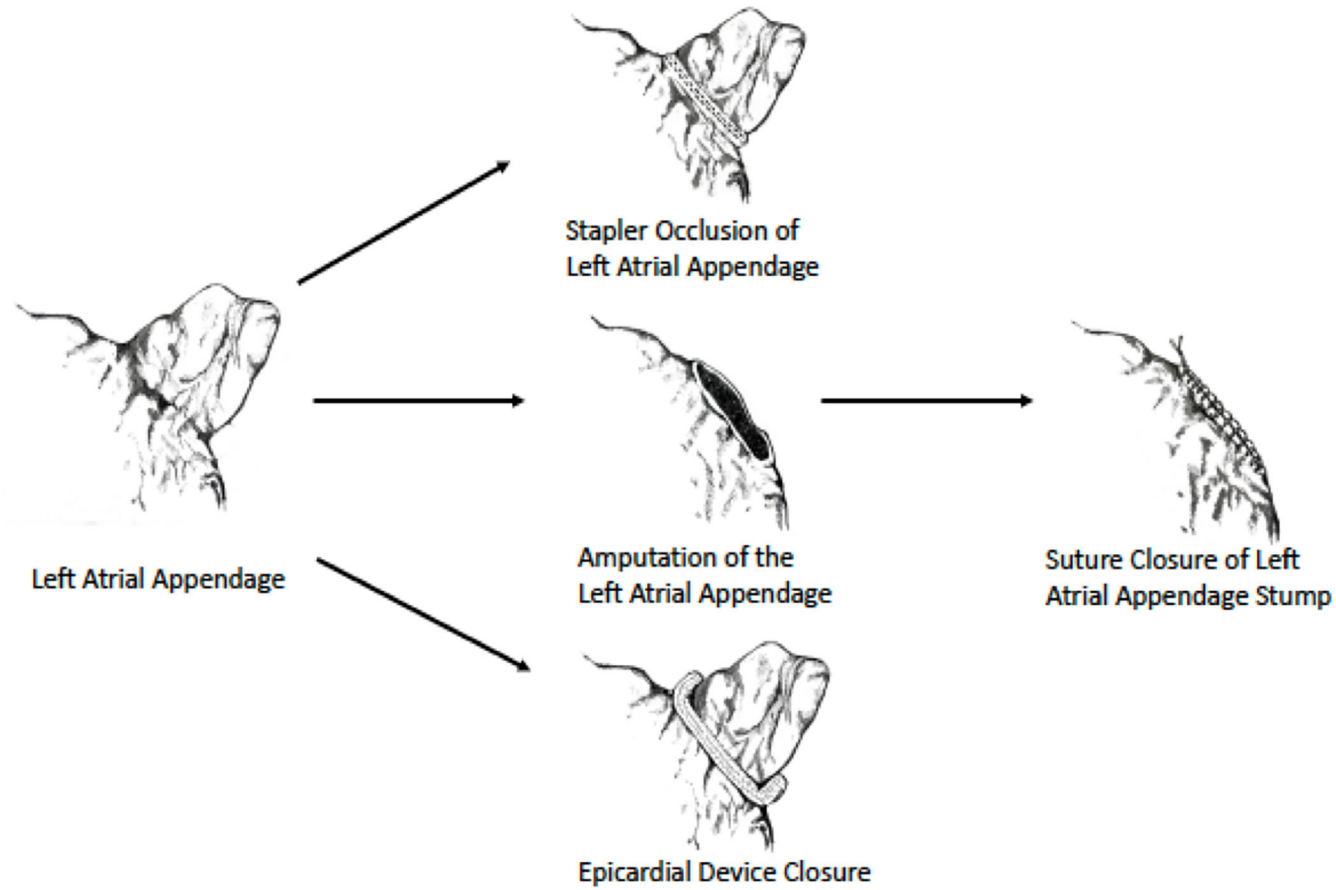
Surgical LAA occlusion techniques used in LAAOS III trial. Image reprinted with permission from the New England Journal of Medicine (52).

As data regarding surgical LAA exclusion were until recently derived mainly from non-randomized studies, current society guidelines provide a relatively “weak” recommendation (class IIb) for performing it during cardiac surgery (13, 50) (Table 1). However, the results of the LAAOS III trial (51, 52), which were recently published, could potentially change the current practice. In this large, multicenter randomized trial, 4,811 patients who underwent cardiac surgery and had AF and CHA_2_DS_2_ VASc scores of ≥2 were randomized to receive standard procedure or to undergo concomitant LAA occlusion by one of the following techniques: amputation and closure (most commonly), stapler closure, double-layer linear closure, or closure with an approved epicardial exclusion device (Figure 1). Most patients continued anticoagulation therapy after surgery. The study was stopped early after a prespecified interim analysis of efficacy. At 3-year follow-up, compared with patients who received standard care, those who underwent LAA occlusion had a 33% lower risk for stroke or systemic embolism [4.8% vs. 7.0% (HR 0.67, 95% CI 0.53–0.85)]. No difference was observed in the rates of death, heart failure hospitalization, myocardial infarction, and perioperative bleeding, providing reassuring data alleviating potential concerns regarding the safety of the concomitant LAA closure during cardiac surgery.

**Table 1:** Comparison of European vs. American guidelines on left atrial appendage occlusion/exclusion.

	**2020 ESC guidelines for the diagnosis and management of AF**		**2019 AHA/ACC/HRS focused update on the management of patients with AF**	
Percutaneous LAA occlusion	May be considered in patients with AF and contraindications for long term OAC therapy		May be considered in patients with AF and contraindications for ng term OAC therapy	
	COR: IIb	LOE: B	COR: IIb	LOE: B-NR
Surgical LAA occlusion/exclusion	Surgical occlusion or exclusion of the LAA may be considered in patients with AF undergoing cardiac surgery.		Surgical occlusion of the LAA may be considered in patients with AF undergoing cardiac surgery.	
	COR: IIb	LOE: C	COR: IIb	LOE: B-NR

ACC, American college of cardiology; AHA, American heart association; AF, atrial fibrillation; COR, class of recommendation; ESC, European society of cardiology; HRS, heart rhythm society; LAA, left atrial appendage; LOE, level of evidence; NR, nonrandomized; OAC, oral anticoagulation.

COR-IIb is defined as usefulness/efficacy is less established by evidence/opinion (European guidelines), benefit ≥ risk (American guidelines).

LOE-B is determined when data derived from a single randomized clinical trial or large non-randomized studies (European guidelines), and B-NR when evidence derived from 1 or more well-designed, well-executed non-randomized studies, observational studies or registry studies (American guidelines).

LOE-C is determined as consensus opinion of experts and/or small studies, retrospective studies, registries (European guidelines).

A few limitations of the study should be noted. First, there was no comparison of efficacy between the different LAA occlusion techniques that were used. Second, the authors did not perform a systematic echocardiographic surveillance for LAA occlusion failure, neither after cardiac surgery nor in patients with recurrent stroke.

The LAAOS III trial results strongly support an add-on surgical exclusion of the LAA during otherwise needed cardiac surgery as a complementary measure to oral anticoagulation therapy in AF patients with CHA_2_DS_2_ VASc of ≥2 (52).

### Percutaneous left atrial appendage exclusion techniques

Another approach, using a Lariat device (SentreHEART, Inc., Redwood City, California), combines both percutaneous endocardial and epicardial access for LAA exclusion. The procedure involves percutaneous deployment and attachment of magnet-tipped wires in the endocardial and epicardial surfaces of the LAA and using them as a rail for epicardial advancement of the Lariat device to the base of the LAA, where a pre-tied suture is released achieving occlusion of the LAA by complete ligation of the LAA neck. The largest Lariat registry of 712 patients at 18 US hospitals reported a success rate with device deployment of over 95%, peripocedural complications rate of ~5%, and delayed pericardial complications rate of 4.8% (53). It should be noted, however, that other reports described higher periprocedural complications as well as post-procedural leak rates (54, 55). The Lariat device received European CE mark approval in 2015 for LAA occlusion (56). However, the FDA approved the device for soft tissue closure but not LAA occlusion, so its use in the US for this purpose is off-label (57). As an epicardial device with no direct contact with blood and a minimally invasive implantation technique, the Lariat device may serve as a therapeutic option for frail patients with absolute contraindications for any antithrombotic therapy (56).

#### The trans-catheter techniques

**Figure 2:**
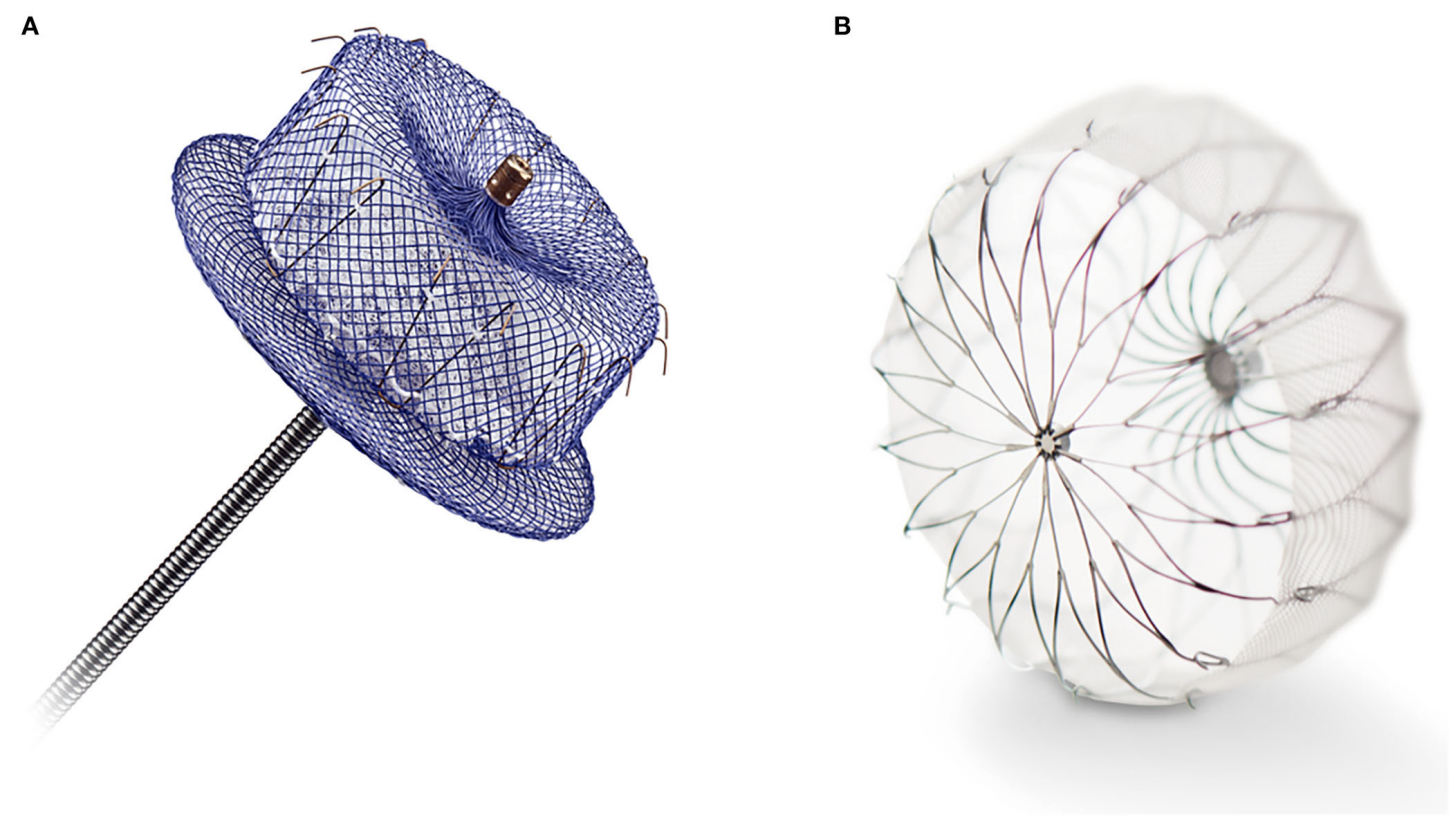
**(A)** The Amplatzer AMULET Left Atrial Appendage Occluder (Abbott, Abbott Park, Illinois, USA). **(B)** The WATCHMAN FLX LAA system (Boston Scientific, Marlborough, Massachusetts, USA).

Both interest and skill with percutaneous techniques for structural heart procedures have driven the development of minimally invasive LAA exclusion using the trans-catheter approach (58). To date, several devices have been designed explicitly for LAA occlusion using the trans-catheter approach. The most utilized two devices are the Amplatzer AMULET Left Atrial Appendage Occluder (Abbott, Abbott Park, Illinois, USA) and the WATCHMAN and WATCHMAN FLX LAA system (Boston Scientific, Marlborough, Massachusetts, USA) (Figure 2).

Pre-procedural evaluation of the LA and LAA for all the devices, including excluding thrombus, verification of placement, and evaluation of post-procedural pericardial effusion, requires skilled fluoroscopic and TEE/ICE coordination (27). Computed tomography (CT) can be used as an assistant for pre-operative assessment of the LAA shape, size, and planning of an appropriate LAA occluding device placement (26). Cardiac magnetic resonance may offer some imaging advantages and help select the device's type and size (59, 60), but its role in the pre-procedural planning stage is yet to be determined. All systems are delivered percutaneously through trans-septal access to the LA.

##### Watchman device

The Watchman device is currently the most commonly used percutaneous LAA occlusion device. The Watchman LAA occluding device is based on a self-expanding nitinol frame structure with a row of fixation barbs, though it is covered with a polyethylene membrane, being permeable only at the side of the LA (61). This device has been evaluated in a randomized trial—Watchman Left Atrial Appendage System for Embolic Protection in Patients with Atrial Fibrillation (PROTECT-AF) (26). This study was designed to assess the non-inferiority of the device against chronic anticoagulation therapy with warfarin. Patients undergoing Watchman implantation received warfarin for a minimum of 45 days after the procedure, which was discontinued if 45-day TEE showed satisfactory LAA closure (<5 mm wide residual jet of peri-device color Doppler flow). After warfarin treatment was stopped, once-daily clopidogrel (75 mg) and aspirin (81–325 mg) were prescribed until a 6-month follow-up visit, then aspirin alone was continued indefinitely (26). Efficacy was assessed by a primary composite endpoint of stroke, cardiovascular death, and systemic embolism. At 1,065 patient-years of follow-up, the trial showed that the efficacy of percutaneous closure of the LAA using the Watchman device was non-inferior to that of chronic Warfarin therapy (26). Primary safety events (events related to excessive bleeding, for example, intracranial or gastrointestinal bleeding, or procedure-related complications, for example, severe pericardial effusion, device embolization, procedure-related stroke) occurred at a higher rate in the intervention group than in the control group (RR 1.69, 95% CI 1.01–3.19) with up to 5% of procedure-related significant pericardial effusion accumulation, which required percutaneous or surgical drainage. No patients with pericardial effusion died or suffered from long-term disability, although the length of hospital stay for these patients was longer than in patients without a pericardial effusion (median 4 days longer). Effusion rates declined with investigator experience during the study course.

The influence of the operator's experience on the safety of percutaneous LAA closure was assessed in an analysis of patients from the PROTECT-AF trial who underwent attempted device LAA closure (*n* = 542) and from a subsequent non-randomized registry of patients undergoing Watchman implantation [Continued Access to PROTECT-AF (CAP) Registry; *n* = 460] (62). There was a significant decline in the rate of procedure or device-related safety events between the first and second halves of PROTECT-AF and CAP, with 10.0, 5.5, and 3.7% of patients, respectively, experiencing events (*P* = 0.006). The rate of serious pericardial effusion decreased from 5.0% in PROTECT-AF to 2.2% (*p* = 0.019) in the CAP registry, whereas periprocedural stroke decreased from 0.9 to 0% (*p* = 0.039) (63, 64).

Several concerns were raised by the US Food and Drug Administration (FDA) regarding patient selection criteria (e.g., the inclusion of patients with CHADS2 score of 1) and acute safety events, particularly in the early portion of the trial, and a second trial was recommended. A second randomized pivotal trial [PREVAIL (Evaluation of the WATCHMAN LAA Closure Device in Patients With Atrial Fibrillation Versus Long Term Warfarin Therapy); NCT01182441] aimed at documenting continued improved safety and at confirming the efficacy demonstrated in the PROTECT AF trial (63). Importantly, the CHADS_2_ score of the study population was higher than in PROTECT-AF, and as pre-specified, 38.8% of patients were enrolled at new sites and 39.1% of procedures were performed by new operators.

The 18-month event rates of the first primary efficacy endpoint [ischemic or hemorrhagic stroke, systemic embolism (SE), and cardiovascular or unexplained death] were similar and low in both the device group and the control group [0.064 vs. 0.063, respectively (RR 1.07, 95% CI 0.57–1.89)]. The upper bound of the confidence interval of 1.89 was not lower than the pre-specified non-inferiority margin of 1.75 predefined in the statistical analysis plan. Thus, statistical non-inferiority was not achieved (63). The rate for the second co-primary efficacy endpoint (stroke or SE >7 days' post-randomization) achieved non-inferiority [0.0253 vs. 0.0200 respectively (RR 0.0053, 95% CI −0.0190 to 0.0273)]. The study showed encouraging results in terms of safety, with 2.2% of significant periprocedural complications [defined as acute (within 7 days) occurrence of death, ischemic stroke, systemic embolism, and procedure/device-related complications requiring major cardiovascular or endovascular intervention]. Of notice, the procedural safety has improved from the earlier PROTECT-AF trial, even though more than a third of the patients underwent the procedure at new sites and with new operators (63).

In a meta-analysis using combined data from the PROTECT-AF and PREVAIL trials, as well as from the CAP and CAP2 (Continued Access to PREVAIL) non-randomized registries that accompanied those trials (*n* = 2,406 with 5,931 patient-years), the Watchman device did not show significant reduction of the composite efficacy endpoint compared with warfarin therapy (HR 0.79, *p* = 0.22) (65), although it should be noted that these studies were designed and powered to detect non-inferiority, not superiority, of the device compared to anticoagulation. Interestingly, however, when the individual components of this composite outcome were analyzed, significant reductions in hemorrhagic stroke (HR 0.22, *p* = 0.004), cardiovascular/unexplained death (HR 0.48, *p* = 0.006), and major non-procedural-related bleeding (HR 0.51, *p* = 0.002) were observed in Watchman implanted patients compared with warfarin treatment. This observation was counterbalanced with a higher rate of ischemic stroke or systemic embolism (HR 1.95, *p* = 0.05) in the Watchman group, which could be potentially explained by technical failures of the device, including failure to completely obliterate LAA flow, anatomical remodeling of the LAA ostium post-implantation leading to more leaks, and thrombus formation on the device (65).

The Watchman device which received a Conformité Européenne (CE) mark in 2005 (65) was approved by the FDA in March 2015 for use in non-valvular (i.e., no prosthetic mechanical valve or moderate-severe mitral stenosis) AF patients to reduce the risk of stroke. Following FDA approval, the Centers for Medicare and Medicaid Services (CMS) approved coverage of Watchman device implantation in non-valvular AF patients with CHA_2_DS_2_-VASc score ≥3 who are considered as high-risk for bleeding but can take short-term OAC therapy. Globally, insurance coverage policies vary, affecting the LAAO procedures rate. For instance, the Watchman device was approved for use in China in 2013, and by 2017, there was a relatively low rate of ~2,000 implantations annually (66). Moreover, a recently published US study from the NIS database showed that minority patients undergoing Watchman implantation had a higher burden of key comorbidities and also experienced increased Watchman-related procedural complications (67).

In an early report after FDA approval, a total of 3,822 patients were implanted with a Watchman device by 382 operators, many of whom were new and inexperienced, in different centers across the US. A 95.6% success rate was observed with a relatively low complications rate compared with previous studies including 39 (1.02%) pericardial tamponades, 9 device embolizations (0.24%), and 3 procedure-related strokes (0.078%). However, it should be noted that three procedure-related deaths occurred (0.078%) were observed to be due to cardiac tamponade (68).

Ledesma et al. recently published an analysis of the frequency and timing of adverse events associated with an estimated total of 43,802 Watchman device implantations performed after the FDA approval in 2015. Using the FDA Manufacturer and User Facility Device Experience (MAUDE) real-world database, the authors reported an overall adverse events rate of 7.3%, most commonly pericardial complications (1.6%), and a mortality rate of 0.4%. Interestingly, while most adverse events occurred within 1 day of the procedure, a significant proportion of device embolizations, strokes, and deaths occurred >1-month post-implantation (69).

Long-term data from the Watchman pivotal trials and respective registries were published more recently. A meta-analysis of a 5-year follow-up data from the PROTECT-AF and PREVAIL trials (*n* = 1,114) showed that compared with warfarin treatment, LAA closure was associated with a significant decrease in hemorrhagic stroke (HR 0.20, *p* = 0.0022), cardiovascular/unexplained death (HR 0.59, *p* = 0.027), and all-cause death (HR 0.73, *p* = 0.035) (70). Noticeably, a trend toward more ischemic strokes and SE was associated with the device implantation (HR 1.71, *p* = 0.08). However, a recent study of long-term outcomes of CAP and CAP2 registries of Watchman implanted patients showed relative reductions of 78 and 69% in ischemic stroke rate, respectively, compared to the predicted rate according to their CHA_2_DS_2_-VASc scores (71).

PROTECT-AF, PREVAIL, and their accompanying CAP and CAP2 registries had similar enrollment criteria and required anticoagulation therapy for all participants. Evidence on safety and efficacy for Watchman implanted patients who are unable to take OAC stems from case series and registries. The ASA Plavix Feasibility Study with WATCHMAN Left Atrial Appendage Closure Technology (ASAP) study was a non-randomized prospective registry of 150 patients with a CHADS_2_ score ≥1 and a mean CHA_2_DS_2_-VASc score of 4.4, who were ineligible for OAC (24). A dual antiplatelet therapy regimen of clopidogrel and aspirin for 6 months followed by aspirin alone indefinitely was commenced at Watchman implantation procedure. The annual ischemic stroke rate was 1.7% which was 77% lower than the expected annual rate of 7.3% based on the CHADS_2_ score of the patient cohort (24). The EWOLUTION registry is a multicenter prospective non-randomized cohort study, which enrolled 1,025 patients who received a Watchman device. About 73% of patients were deemed unsuitable for OAC therapy and had a mean CHA_2_DS_2_-VASc score of 4.5 ± 1.6 (72). After 1-year follow-up, 1.1% of patients had an ischemic stroke which translates into 84% risk reduction compared with the expected stroke rate based on CHA_2_DS_2_-VASc score (72).

The National Cardiovascular Data Registry (NCDR) LAAO is a national post-marketing surveillance program prospectively following the clinical results of 38,158 Watchman device implantation procedures performed in 495 centers in the US (73). Most patients in this registry (69%) had relative or absolute contraindications for OAC therapy. The mean CHA_2_DS_2_-VASc score was 4.6 ± 1.5. Recently, encouraging short-term outcomes were published including a 98.3% implant success rate and in-hospital major adverse events rate of 2.1% less frequent than reported in the pivotal trials (73).

The ASAP-TOO trial [The Assessment of the Watchman Device in Patients Unsuitable for Oral Anticoagulation (NCT02928497)] is an ongoing multicenter prospective randomized trial that aims to assess the safety and efficacy of the Watchman device in patients deemed ineligible for OAC therapy (74). Control patients will be treated with a single antiplatelet agent or no therapy based on physician discretion. Long-term outcomes of the NCDR LAAO registry and the ASAP-TOO trial, when available, will help refine patient selection and establish the role of percutaneous LAA closure using the Watchman device in patients deemed ineligible for long-term OAC therapy.

Recently, the Watchman FLX, a second-generation Boston Scientific LAA occluder device, was introduced. The Watchman FLX is a single-lobe occluder with a nitinol frame and a permeable polyester membrane. It has 36 fixation anchors instead of 10 in the first generation device and comes in a broader range of dimensions (20–35 mm instead of 21–33 mm in the older device). In a prospective, non-randomized, multicenter trial, the effectiveness and safety of the Watchman FLX were evaluated (75). Four hundred patients with a mean CHA_2_DS_2_-VASc score of 4.2 ± 1.5 and 100% had reached the primary effectiveness endpoint of peri-device leak ≤ 5 mm, assessed by the echocardiography at 12-month follow-up (*p* < 0.0001). The primary safety endpoint was reached in 0.5% of patients (*p* < 0.0001). The researchers reported device-related thrombus in seven patients. Three patients (0.7%) had pericardial effusion requiring pericardiocentesis at 45 days and four patients (1%) at 12 months (75). No device embolization was reported. This device has double row stabilizing anchors and distal rounded-edged, which may be leading to this improvement in safety. Comparing the Watchman FLX device to long-term anticoagulation medical therapy with DOACs is now underway in the enrolment of patients in the CHAMPION-AF study (NCT04394546). This study is designed as a non-inferiority trial for the occurrence of stroke, CV death, and SE, and as a superiority trial for non-procedural bleeding for the Watchman FLX arm compared with the DOAC arm. The Watchman FLX device received FDA approval in July 2020 and CE Mark in March 2019, and has largely replaced the earlier Watchman device for new implantations in clinical practice.

##### The amplatzer cardiac plug device

Another device used for LAA occlusion is the Amplatzer Cardiac Plug (ACP) device, a self-expanding, fully retrievable, and repositionable device constructed from a nitinol mesh and polyester patch, and developed based on the Amplatzer double-disk septal occluders (76). The published initial clinical experience from 10 European centers showed that the ACP device was successfully implanted in 132 (96.4%) of 137 patients in whom the procedure was attempted. Despite being performed by highly skilled operators, serious periprocedural complications were reported in 10 of the patients (7%), including ischemic stroke in three, device embolization in two (recaptured and percutaneously removed successfully in both cases), and significant pericardial effusions in five (76). Interim data from the ACP European-market registry showed similar procedural success (96.5%) with a periprocedural complications rate of 5.5%, including three cases of device embolization during the implant procedure (all early in the learning curve of the operators) (76). It is crucial to note that dual antiplatelet therapy with Aspirin and Clopidogrel for 1 to 3 months post-implantation was recommended, as reports raised concern for thrombus formation on the device (76, 77).

As to the efficacy in stroke prevention, early results from the initial Asia-Pacific experience were promising. The ACP device was successfully implanted in 95% of the 20 patients with a mean CHADS_2_ score of 2.3, with no strokes recorded during a mean follow-up of 12.7 ± 3.1 months (78).

The second generation of the ACP, known as the Amplatzer Amulet Left Atrial Appendage Occluder, includes a wider lobe and more stabilizing wires for improved stability and the ability to close larger appendages. The Amulet IDE study randomized 1,878 patients with AF and a mean CHA_2_DS_2_-VASc of 4.6 to receive either the Amulet or the old generation Watchman device (79). The rate of ischemic stroke or systemic embolism and major bleeding, and all-cause death were similar between the two devices. A residual jet of <5 mm was achieved more frequently with the Amulet device but also had more pericardial effusions requiring pericardiocentesis (79). A real-life multicenter registry of 1,088 patients with non-valvular AF reported successful device implantation in 99.0% of patients, while the TEE follow-up in 673 patients showed adequate appendage occlusion in 98.2% of patients (80). The Amulet device received the European CE Mark approval in 2013 and FDA approval in 2021.

Recently, Della Rocca et al. published a meta-analysis of 21 LAAO studies (n=4,186), in which 3,187 patients received an Amulet and 999 Watchman FLX. The mean overall CHA_2_DS_2_-VASc score was 4.3 and was not significantly different between the groups. The authors report that LAAO with Amulet was associated with a significantly higher incidence of periprocedural complications (4.6 vs. 0.6 *p* < 0.01) driven mainly by pericardial and major/intracranial bleeding, as well as a higher incidence of per device leaks >5 mm (0.34 vs. 0.01%, *p* = 0.06) compared with FLX. These data could potentially be more reflective of contemporary clinical practice as the old-generation Watchman device has been replaced by the new-generation Watchman FLX (81).

The PRAGUE-17 study (Left Atrial Appendage Closure vs. Novel Anticoagulation Agent Atrial Fibrillation) (82, 83) was a randomized, non-inferiority comparison of LAA closure vs. DOACs. Four hundred and fifteen patients with non-valvular AF and CHA_2_DS_2_-VASc ≥3 and HAS-BLED ≥2, were enrolled in 10 centers in the Czech Republic and were randomized to DOAC (mostly apixaban) or percutaneous LAA closure. About 61% of implanted devices were Amulet and the rest Watchman or Watchman-FLX. The primary outcome was a composite of stroke, TIA, cardiovascular death, major or non-major clinical relevant bleeding, or procedure/device-related complications. After a median follow-up of 3.5 years, LAA closure achieved non-inferiority vs. DOAC therapy for the primary composite endpoint (HR 81, 95% CI 0.56–1.18) (83). Also, non-procedural bleeding diverged about 6 months after the procedure with more events in the DOAC group [40 vs. 23 (HR 0.55, 95% CI 0.31–0.97)]. It should be noted, however, that the periprocedural complications rate was 4.5% including two procedure-associated deaths (82). In addition, it is important to note that as the trial was underpowered for evaluation of individual components of the primary composite outcome, the study design combined both safety and efficacy events with the potentially competing directions of effects in the composite outcome, and by that making interpretation of the study results more difficult. The Clinical Trial of Atrial Fibrillation Patients Comparing Left Atrial Appendage Occlusion Therapy to Non-vitamin K Antagonists [CATALYST (NCT 04226547)] will evaluate the safety and effectiveness of LAAC with Amulet device compared with DOACs in an adequately powered sample of >2,600 patients with an indication for long-term OAC. This study is designed as a non-inferiority trial for the occurrence of stroke, CV death, and SE, and as a superiority trial for non-procedural bleeding for the Amulet arm compared with the DOAC arm.

##### Post-implantation antithrombotic treatment

Device-related thrombosis (DRT) occurs in 2% to 5% of patients receiving an LAAO device. This number varies with device type and the timing of the post-procedural imaging (26, 27, 62, 78, 80, 84). Although not uniformly defined or classified (56), DRT can be detected by means of TEE or CT with equivalent efficacy (85) and has been associated with ischemic events and major adverse cardiac events (86). A recently published European expert consensus paper recommended that post-procedural imaging to assess for DRT should be done 6 to 24 weeks following implantation and that if DRT is identified on the atrial side of the device, it should be treated with anticoagulation for thrombus resolution (56). A peri-device leak device of more than 5 mm indicates continued oral anticoagulation or a re-do procedure.

Management of antithrombotic treatment post-LAA occlusion remains controversial as society guidelines do not have clear-cut recommendations (13, 87). Treatment protocols from major trials are used today in everyday practice. For Watchman patients with low bleeding risk, OAC (with either Warfarin or DOACs) is started together with Aspirin (75–325 mg QD, indefinitely) after the procedure for 45 days or continued until adequate LAA sealing is confirmed. After that, OAC is discontinued, and Clopidogrel (75 mg QD) is added to Aspirin for 6 months (84). In Watchman patients with high bleeding risk, OAC is not used, and Clopidogrel is used in adjunct to Aspirin for 1–6 months based on adequate LAA sealing (24), although this is currently off-label. The high bleeding risk protocol was suggested for all patients receiving ACP/Amulet devices. Based on a relatively small non-randomized series and expert opinion, a short course of 2–4 weeks of single antiplatelet therapy was suggested for patients considered at extremely high risk for bleeding (56). Patients ineligible even for a such abbreviated period of antiplatelet therapy may be considered for epicardial trans-catheter approach (Lariat device) or surgical LAA exclusion.

Interestingly, a recently published large multicenter post-procedural DRT registry study showed that non-paroxysmal AF, renal insufficiency, hypercoagulability disorders, iatrogenic pericardial effusion, and deep device implantation (>10 mm from the pulmonary vein ridge) were associated with DRT detection during follow-up, while the type of post-procedural antithrombotic regimen employed was not (86).

As current guidelines leave room for operator discretion, we believe that a post-procedural antithrombotic regimen should be tailored on a case-to-case basis, preferably in collaboration with a specialist of the organ involved in the contraindication for OAC.

##### Summary

The well-recognized limitations of vitamin K antagonists led to the pursuit of other pharmacological and non-pharmacological tools to better address the issue of stroke prevention in AF patients. As described earlier, the surgical approach to the LAA exclusion for stroke prevention in AF patients could potentially become standard of care add-on therapy for eligible patients who undergo cardiac surgery, based on the results from the recently published randomized LAAOS III trial. Watchman LAAO device implantation has been the first percutaneous LAA occlusion approach, evaluated in a randomized trial, showing non-inferiority in stroke prevention compared to warfarin therapy. The peri-procedural complication rate was associated with operators' experience, with a learning curve leading to lower rates of adverse events in recent years. Later, a growing body of evidence from prospective studies and registries has shown favorable results for both Watchman and Amulet device implantation as an alternative for stroke reduction in AF patients.

The introduction of DOACs over a decade ago has significantly shifted the risk-benefit ratio of anticoagulation therapy in AF patients, achieving improved efficacy with significantly reduced risk for the most feared complications of intracranial hemorrhage and hemorrhagic stroke. In the absence of large randomized trials comparing the invasive approaches and the DOAC therapy, we are left to make a logical deduction from the available data. At this point, for the majority of patients with CHA_2_DS_2_-VASc score of 2 or above who do not have any contraindication to anticoagulation, the option of relatively safe, effective, and convenient DOAC pharmacotherapy, not involving any operator skills or exposure to the risk of invasive procedure, seems favorable. However, the LAA exclusion remains an important tool in patients, especially in those with contraindication to anticoagulation, in whom a catheter-based approach is a pertinent alternative, with improved safety in recent years.

Although much evidence has been accumulated in favor of LAA occlusion in eligible patients, there are still gaps in the knowledge. First, there is a need for adequately powered randomized trials assessing LAA closure vs. DOAC therapy. Second, catheter-based approaches, as well as surgical approaches for LAA closure, were not well validated in patients with contraindications for anticoagulation. Third, the most appropriate post-procedural/surgical antithrombotic regimen(s) still needs to be elucidated. In view of these gaps in the evidence, European and American practice guidelines give a relatively “weak” IIb indication for LAA occlusion procedures (Table 1). In the coming years, growing evidence from ongoing trials on percutaneous LAAC will potentially substantiate our knowledge of this therapeutic approach, and this may eventually translate into a change in practice guidelines.

## Author contributions

GR and GE-G conceived the idea and design of the study and drafted the paper. GM, ER, and IM collected the data. AR, DP, IT, JR, and MM provided revisions to the manuscript. OA and EH provided revisions and principle investigators. All authors contributed to the article and approved the submitted version.

## Conflict of interest

The authors declare that the research was conducted in the absence of any commercial or financial relationships that could be construed as a potential conflict of interest.

## Publisher's note

All claims expressed in this article are solely those of the authors and do not necessarily represent those of their affiliated organizations, or those of the publisher, the editors and the reviewers. Any product that may be evaluated in this article, or claim that may be made by its manufacturer, is not guaranteed or endorsed by the publisher.
